# PReS13-SPK-1129: Biologic therapies in SLE

**DOI:** 10.1186/1546-0096-11-S2-I6

**Published:** 2013-12-05

**Authors:** DA Isenberg

**Affiliations:** 1 Rheumatology, University College London/University College Hospital London, London, UK

## 

Biologic therapy has not made the breakthrough in the treatment of patients with systemic lupus erythematosus (SLE) that it has managed in patients with rheumatoid arthritis and psoriatic arthritis. However, there are distinct signs that this form of treatment is going to find a place in the pantheon of SLE therapy. Based on a better knowledge of the ethiopathogenesis of lupus drugs, which interfere with B cell activation notably, Benlysta and Atacicept have been reported in major clinical trials to be successful in terms of controlling disease activity and preventing flares respectively. In addition, although the formal clinical trials did not meet their primary endpoints (almost certainly because of problems with the designs of those trials) the use of B cell depletion in the form of rituximab is widely accepted and appears to be helpful and highly beneficial in aspects of lupus ranging from skin disease to severe proliferative glomerulonephritis.

My colleagues and I have treated 120 lupus patients with rituximab and have reported that 90% of patients are in partial or complete remission at six months (Lu et al. Arthritis Care Res; 2009: 61, 482), though some questions remained unanswered. For example, why is it in some patients B cells return within two months and in other cases it may be many years? In addition, once the B cells have returned there is a highly variable length of time before the clinical features return. In spite of disappointing results with major clinical trials, at least two new clinical trials using rituximab, are about to start recruiting, the RING trial for patients with lupus nephritis who failed conventional therapy at six months and RITUXILUP which aims to treat lupus patients at the time of diagnosis with two infusions of rituximab followed by a low dose of mycophenolate the state of aim avoiding the use of oral steroids. In an open label study of 50 lupus nephritis patients given rituximab at the time of diagnosis followed by low dose mycophenolate, Condon et al (Ann Rheum Dis 2013. In press) have reported that just two out of 50 patients after two years have required oral steroids.

## Disclosure of interest

None declared.

